# A Cost-Effective Treatment Approach Using Rosuvastatin+Clopidogrel Fixed Dose Combination (FDC) to Enhance Adherence: A Retrospective, Non-comparative, Real-World Indian Study

**DOI:** 10.7759/cureus.89471

**Published:** 2025-08-06

**Authors:** Kishor Pargaonkar, Sanjeev Chaudhary, Arijit Roy Choudhury, Anil Samaria, Sudhir Shendage, Ganesh Narayana, Ashootosh Bhardwaj, Anil Balachandran, Ranga Reddy BVA, Pruthviraj Sinh Puwar, Nutan Kumar, Snehal Bansode, Abhijit Pednekar

**Affiliations:** 1 Cardiology, Pargaonkar Hospital, Aurangabad, IND; 2 Cardiology, Marengo Asia Hospitals, Gurgaon, IND; 3 Medicine, Heilen Cross, Kolkata, IND; 4 Medicine, Jawaharlal Nehru Medical College, Ajmer, IND; 5 Internal Medicine, Arogyam Healthcare, Mumbai, IND; 6 Cardiology, Dhanalakshmi Srinivasan Medical College and Hospital, Trichy, IND; 7 Cardiology, Bhardwaj Heart Care, Chandigarh, IND; 8 Cardiology, Lakshmi Hospital, Ernakulam, IND; 9 Cardiology, Apollo Hospitals, Hyderabad, IND; 10 Cardiology, Jupiter Hospital, Vadodara, IND; 11 Cardiology, Tulip Heart Centre, Anand, IND; 12 Cardiology, Seven Hills Hospital, Bangalore, IND; 13 Scientific Services, USV Private Limited, Mumbai, IND

**Keywords:** adherence, cardiovascular disease, clopidogrel, efficacy, rosuvastatin, tolerability

## Abstract

Background

Medication adherence is mostly influenced by cost, and disease management can be achieved through cost-effective combinations. The present study aimed to evaluate adherence to the cost-effective fixed dose combination (FDC) of rosuvastatin and clopidogrel in the management of cardiovascular diseases (CVD).

Methods

This retrospective, non-randomized, non-comparative, multicenter study was conducted across 100 healthcare centers in India. Patients aged ≥18 years, of either sex, who were prescribed a combination of rosuvastatin and clopidogrel, and had a clinical diagnosis of atherosclerotic cardiovascular disease (ASCVD) or were post-acute coronary syndrome (ACS) or were at high risk for cardiovascular events, as determined by standard care practices in medicine and cardiology, were included in the study.

Results

A total of 975 patients were included in the study. Hypertension was the most common comorbidity observed in 749 (76.82%) patients, followed by diabetes in 706 (72.41%) patients. All patients received a clopidogrel dose of 75 mg. However, 492 (50.46%) patients were prescribed rosuvastatin 20 mg, while 483 (49.54%) patients received the 10 mg dose. Adherence to all prescribed doses was reported in 931 patients (95.49%). The most commonly reported reasons for treatment adherence with clopidogrel and rosuvastatin were decreased cost, reported by 672 patients (68.92%), and simplified dosing, reported by 589 patients (60.41%). Furthermore, 548 (56.21%) physicians rated the efficacy of a combination of clopidogrel and rosuvastatin as excellent, while 549 (56.31%) physicians rated its tolerability as excellent. Better adherence and treatment of dyslipidemia were significantly more common reasons for prescribing treatments among patients with an income of >10 lakh compared to those with an income of <5 lakh and five to 10 lakh (P=0.023 and P=0.047, respectively). The physician’s global evaluations of both efficacy and tolerability were rated as “Excellent” significantly more commonly in patients receiving the 20 mg dose of rosuvastatin compared to those receiving the 10 mg dose (P=0.004 and P=0.028, respectively).

Conclusion

The combination of clopidogrel and rosuvastatin was well-tolerated, with high adherence in patients with ASCVD, post-ACS, and those at high cardiovascular risk.

## Introduction

Cardiovascular diseases (CVD) are chronic, non-communicable diseases that remain the leading cause of morbidity and mortality worldwide [1]. The impact of CVD is profound, resulting in reduced quality of life, loss of life years, and substantial direct and indirect medical expenses [1]. Among CVDs, hypertension is the most common, affecting 20% to 50% of the global adult population. High blood pressure is a well-established risk factor for various serious health conditions, including coronary heart disease (CHD), heart failure, stroke, peripheral arterial disease, kidney failure, and atrial fibrillation [2].

To mitigate cardiovascular events, lipid-lowering agents and antiplatelet therapies play a pivotal role. Among these, rosuvastatin is a statin well-known for its remarkable effectiveness in controlling blood lipid levels [3]. Clopidogrel, an antiplatelet medication, is frequently prescribed to treat acute coronary syndrome (ACS), myocardial infarction, strokes, and peripheral arterial disease [4].

Clopidogrel complements statins for cardiovascular event prevention [5]. The combination of antiplatelet agents and statins is integral to the management of CVDs, with evidence suggesting that such a regimen can significantly reduce the risk of thrombosis [6].

In this context, fixed-dose combinations (FDCs) of statins and antiplatelet agents offer several advantages over free drug combinations. These include improved convenience, better patient adherence, enhanced therapeutic control, reduced treatment costs, and fewer complications. Therefore, the FDC of rosuvastatin and clopidogrel may enhance medication compliance among CVD patients, ultimately contributing to a lower risk of adverse cardiovascular events and overall treatment burden [6].

However, the long-term efficacy of this combined regimen is heavily dependent on patient adherence. The patient’s response to treatment and their commitment to following the prescribed regimen are critical determinants of therapeutic success [7]. Medication non-adherence can undermine this success, leading to elevated mortality rates, worsened health outcomes, and increased healthcare costs [8]. Therefore, ensuring adherence to cost-effective therapy is crucial for achieving the desired clinical outcomes and reducing the burden on healthcare systems. Among lipid-lowering therapies, cost considerations play a crucial role in adherence. In India, the monthly cost of the recommended dose of rosuvastatin ranges from Rs 400 to Rs 800 [9]. Studies suggest that rosuvastatin is more efficacious and cost-effective as compared to atorvastatin in lowering low-density lipoprotein cholesterol and treating patients to target low-density lipoprotein cholesterol levels [10]. Moreover, clopidogrel was also found to be cost-effective as compared to ticagrelor in patients with acute coronary syndrome [11]. The analysis of variation in the cost of hypolipidemic drugs in the Indian market demonstrated a higher cost variation for rosuvastatin 10 mg (429.655 INR) as compared to the FDC of rosuvastatin 10 mg + clopidogrel 75mg (323.076 INR) [12].

Therefore, the present study aimed to evaluate the adherence to cost-effective FDC of rosuvastatin with clopidogrel in patients diagnosed with atherosclerotic cardiovascular disease (ASCVD), post-ACS, and those at high risk of cardiovascular events.

## Materials and methods

Study design

This retrospective, non-randomized, non-comparative, multicenter study was conducted across 100 healthcare centers in India. The study was approved by an Independent Ethics Committee (ACEAS-Independent Ethics Committee, Ahmedabad, EC approval no.: CELE/USV/RWS/2024) and was conducted in accordance with the principles of the Declaration of Helsinki.

Inclusion criteria and exclusion criteria

Patients aged 18 years or older, of either sex, who were prescribed a combination of rosuvastatin and clopidogrel, and had a clinical diagnosis of ASCVD or were post-ACS or were at high risk for cardiovascular events, as determined by standard care practices in medicine and cardiology, were included in the study. Patients having incomplete data files were excluded from the study.

Outcomes

The outcomes of this study were to assess the socioeconomic factors, including affordability and adherence to these medications; the percentage of prescribed doses; and the duration of clopidogrel and rosuvastatin therapy.

The secondary outcomes of this study were to examine the number and percentage of patients with coronary artery disease (CAD) who underwent thrombolysis, percutaneous coronary intervention (PCI), or coronary artery bypass grafting (CABG) while on the clopidogrel and rosuvastatin treatment regimen; to assess the side effect profile over the last six months related to both medications; to record the number and percentage of patients who switched from dual antiplatelet therapy (DAPT) with a statin to the clopidogrel and rosuvastatin combination; and to evaluate the changes in quality of life scores from baseline in both groups to determine the impact of treatment on overall well-being.

Assessment of adherence, affordability, and adverse events

The treatment adherence was documented based on the physician’s assessment of the patient’s dosing behavior and recorded as a binary response (Yes/No) to the question. In cases of non-adherence, the specific reason was selected from predefined categories, including forgot medication once a month, forgot medication two to three times a month, and forgot medication once a week. This method relied on physician-reported adherence based on patient interaction during clinic visits.

Affordability was assessed based on the treating physician’s selection of the reason for prescribing the treatment. One of the predefined options for prescribing rationale was "Better affordability," which allowed physicians to indicate whether cost considerations, such as decreased medication cost or improved cost-effectiveness, influenced the treatment choice. 

Side effects reported in this study were primarily based on patient-reported outcomes and not classified using standardized adverse event grading criteria.

Data collection

Data were collected using electronic case report forms (eCRFs) to record all information required by the study protocol for each participant. The data management team provided access to the eCRFs for the Investigator. Only validated information from source documents was transcribed into the eCRFs.

Data related to demographic characteristics, duration of disease, co-morbidities, concomitant medications, and dosage patterns were collected from medical records authenticated by physicians and cardiologists during routine care. Medical records from various centers treating patients with the rosuvastatin and clopidogrel combination were recorded. Medication data and details on co-morbidities were also gathered.

Statistical analysis

Data were analyzed using SPSS version 23.0 (IBM Corp, Armonk, NY). Missing data within the included patient records were handled using appropriate statistical methods, such as imputation or exclusion, depending on the extent and nature of the missing values. Sensitivity analyses were conducted where applicable to ensure the robustness of the findings. Descriptive statistics were used to describe categorical variables (frequency and percentages) and continuous variables [mean and standard deviation (SD)]. Comparison of quantitative data between two groups was performed using the Mann-Whitney U test, and among more than two groups using the Kruskal-Wallis test. Comparison of quantitative data between the groups was done using the Chi-square test. A P value of <0.05 was considered statistically significant.

## Results

A total of 975 patients were included in the study, of which 694 (71.18%) were men. The median interquartile range (IQR) age of patients was 57 (51.00-63.00) years. The median body mass index (BMI) was 26.96 kg/m². The median systolic blood pressure (SBP) and diastolic blood pressure (DBP) were 140 mmHg and 90 mmHg, respectively. Income data were available for 774 out of the total 975 participants, while data for the remaining 201 were missing due to incomplete documentation in the medical records. Among those with available data, 385 participants (49.74%) had an income of less than five lakh, 313 (40.44%) had an income between five to 10 lakh, and 76 (9.82%) had an income of more than 10 lakh. About 447 (45.85%) patients had smoking habits. About 626 patients had diabetes (64.21%), followed by a family history of dyslipidemia in 626 (64.10%) patients and hypertension in 562 (57.64%) patients. The median duration of CAD and dyslipidemia among the patients was 26 months (Table 1).

**Table 1:** Demographic characteristics IQR: interquartile range; SBP: systolic blood pressure; DBP: diastolic blood pressure; CAD: coronary artery disease.

Parameters	Number of patients (N=975)
Sex, n (%)	
Men	694 (71.18%)
Women	281 (28.82%)
Age (years), median (IQR)	57.00 (51.00-63.00)
Height (cm), median (IQR)	165.00 (157.00-170.00)
Weight (Kg), median (IQR)	72.00 (64.00-80.00)
Body mass index (Kg/m^2^), median (IQR)	26.96 (24.03-29.76)
Blood pressure (mmHg), median (IQR)	
SBP	140.00 (130.00-150.00)
DBP	90.00 (82.00-92.00)
Education level, n (%)	
Educated	746 (76.51%)
Uneducated	229 (23.49%)
Occupation, n (%)	
Employed	267 (27.38%)
Business	234 (24.00%)
Homemaker	162 (16.62%)
Retired	141 (14.46%)
Farmer	132 (13.54%)
Unemployed	39 (4.00%)
Income (Lakh), n (%)	[n=774]
<5	385 (49.74%)
5-10	313 (40.44%)
>10	76 (9.82%)
Place of stay, n (%)	
Urban	727 (74.56%)
Rural	248 (25.44%)
Routine physical activity, n (%)	548 (56.21%)
Smoking habit, n (%)	447 (45.85%)
Family history, n (%)	
Diabetes	626 (64.21%)
Dyslipidemia	625 (64.10%)
Hypertension	562 (57.64%)
Other	40 (4.10%)
Duration of CAD (months), median (IQR)	26.00 (16.00-42.00)
Duration of dyslipidemia (months), median (IQR)	26.00 (16.00-42.00)

Hypertension was the most common comorbidity observed in 749 (76.82%) patients, followed by diabetes in 706 (72.41%) patients, acute coronary syndrome in 667 patients (68.41%), and obesity (37.54%) in 366 patients (37.54%) (Table 2). 

**Table 2:** Medical history or comorbidities Others: GERD (n=15); COPD/asthma (n=11); Hypothyroidism (n=6); Heart failure (n=6); chest discomfort (n=5); Gout and epilepsy (n=4); Anxiety and panic disorder (n=3); Diabetic kidney disease (n=2); Fibroid (n=2). COPD: chronic obstructive pulmonary disease; GERD: gastroesophageal reflux disease.

Parameters	Number of patients (N=975)
Medical history or comorbidities, n (%)	
Hypertension	749 (76.82%)
Diabetes	706 (72.41%)
Acute coronary syndrome	667 (68.41%)
Obesity	366 (37.54%)
Myocardial ischemia	244 (25.03%)
Stroke	171 (17.54%)
Chronic kidney disease	99 (10.15%)
Peripheral arterial disease	55 (5.64%)
Non-alcoholic fatty liver disease	28 (2.87%)
Others	54 (5.54%)

All patients received a clopidogrel dose of 75 mg. However, 492 (50.46%) patients were prescribed rosuvastatin 20 mg, while 483 (49.54%) patients received the 10 mg dose. Around 929 (95.28%) patients took their medications once daily. The median (IQR) treatment duration was 18 months (12.00-30.00). The primary reason for prescribing treatments was to treat or reduce cardiovascular complications in 914 (93.74%) patients. About 377 (39%) patients underwent thrombolysis, percutaneous coronary intervention, or coronary artery bypass graft for CAD while on clopidogrel and rosuvastatin treatment. However, 463 (47.49%) patients switched from a combination of aspirin, clopidogrel, and a statin to a combination of clopidogrel and rosuvastatin during treatment. Adherence to all prescribed doses was reported in 931 (95.49%) patients. The most common reasons for the treatment adherence were decreased cost, reported by 672 (68.92%) patients, and simplified dosing, reported by 589 (60.41%) patients. Out of 975 patients, only 8 (0.82%) patients experienced side effects related to clopidogrel and rosuvastatin treatment in the last six months. Among these eight patients, the most common side effects were nausea and stomach pain, each reported in three patients (Table 3). 

**Table 3:** Treatment patterns IQR: interquartile range; PCI: percutaneous coronary intervention; CABG: coronary artery bypass graft; CAD: coronary artery disease; FDC: fixed dose combination.

Parameters	Number of patients (N=975)
Clopidogrel dose, n (%)	
75 mg	975 (100.00%)
Rosuvastatin dose, n (%)	
10 mg	483 (49.54%)
20 mg	492 (50.46%)
Frequency, n (%)	
Once daily	929 (95.28%)
Twice daily	46 (4.72%)
Treatment duration (months), median (IQR)	18.00 (12.00-30.00)
Reasons for prescribing treatments, n (%)	
To treat/reduce cardiovascular complications	914 (93.74)
Better adherence	591 (60.62%)
To treat dyslipidemia	581 (59.59%)
Better affordability	576 (59.08%)
Patient undergoes thrombolysis/PCI/CABG for CAD while on clopidogrel and rosuvastatin treatment, n (%)	377 (38.67%)
Patients switch from aspirin + clopidogrel (any other anti-platelet) and statin to FDC clopidogrel and rosuvastatin during treatment, n (%)	463 (47.49%)
Patient adherence to all prescribed doses, n (%)	931 (95.49%)
Reason for non-adherence of the doses, n (%)	44 (4.51%)
Once a week	10 (22.73%)
Once a month	16 (36.36%)
Twice a month	15 (34.09%)
Cost-related nonadherences	3 (6.82)
Reasons for treatment adherence with clopidogrel and rosuvastatin, n (%)	
Decreased cost	672 (68.92%)
Simplified dosing	589 (60.41%)
Improved symptoms and health outcomes	549 (56.31%)
Fewer side effects	247 (25.33%)
Other	43 (4.41%)
Patient experienced any side effects related to clopidogrel and rosuvastatin treatment in last 6 months, n (%)	8 (0.82%)
Nausea	3 (37.50%)
Stomach pain	3 (37.50%)
Extreme fatigue	1 (12.50%)
Tiredness	1 (12.50%)
Body aches	1 (12.50%)
Gastritis	1 (12.50%)
Abdomen pain	1 (12.50%)

**Figure 1:**
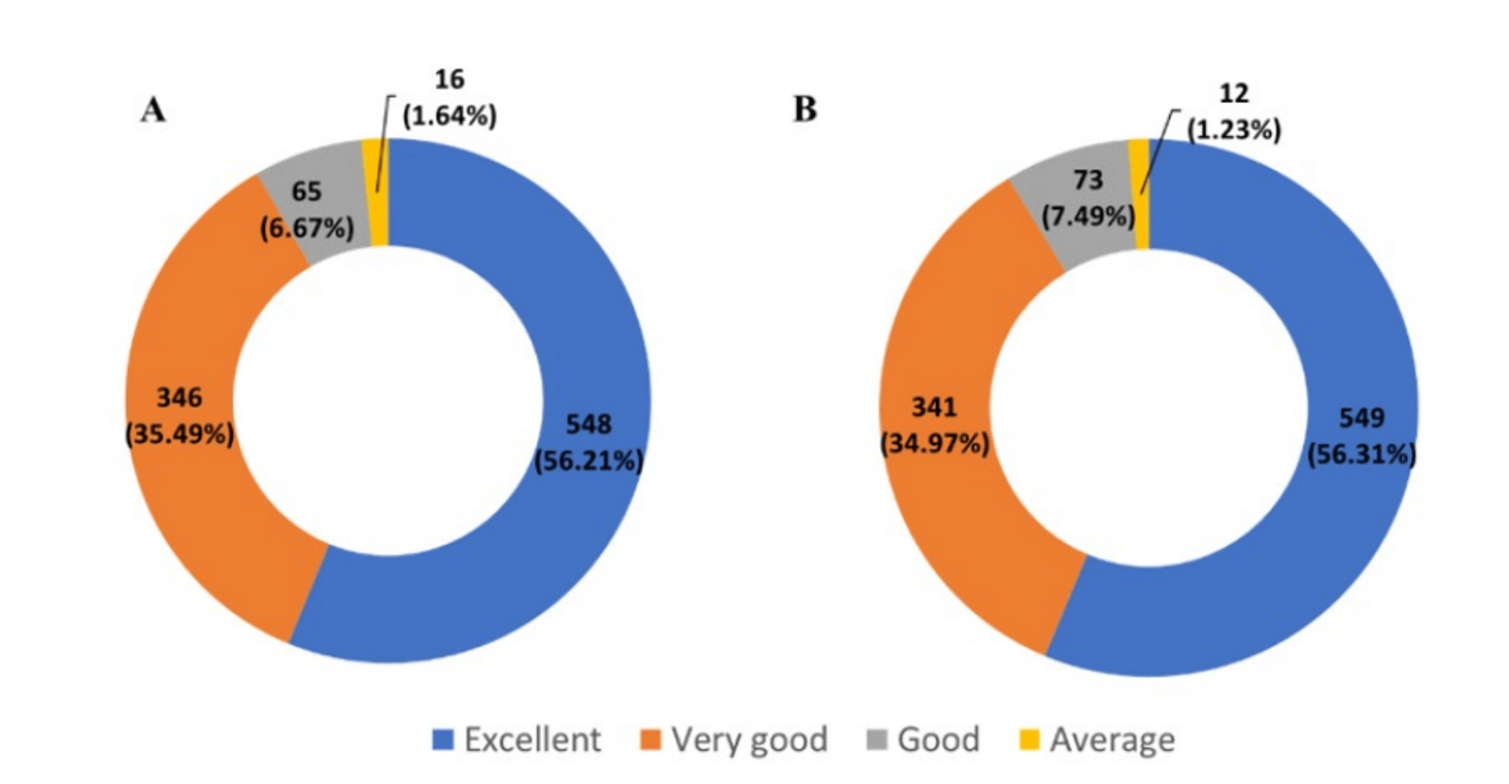
A. Physician global evaluation of efficacy and B. Tolerability

Furthermore, 548 (56.21%) physicians rated the efficacy of a combination of clopidogrel and rosuvastatin as excellent, while 549 (56.31%) physicians rated its tolerability as excellent (Figure 1).

The proportion of patients who received the 20 mg dose of rosuvastatin was significantly higher in the ≥70 years age group compared to the ≥50-<70 years and ≥25-<50 years age groups (62.38% vs 50.15% vs 45.36%; P=0.020). The proportion of patients receiving a once-daily dose was significantly higher in the ≥50-<70 years age group compared to the ≥70 years and ≥25-<50 years age groups (P=0.035). A significantly higher proportion of patients in the ≥50-<70 years age group switched from aspirin + clopidogrel (or another antiplatelet) and statin to FDC clopidogrel and rosuvastatin compared to the ≥25-<50 years and ≥70 years age groups (P=0.001). Physician global evaluations of efficacy and tolerability were rated as 'Excellent' significantly more often in the ≥25-<50 years age group (66.49%) compared to the ≥50-<70 years and ≥70 years age groups (P=0.007 and P=0.006, respectively) (Appendix 1).

A significantly higher proportion of male patients underwent thrombolysis, PCI, or CABG for CAD while on clopidogrel and rosuvastatin treatment compared to female patients (40.92% vs 33.10%; P=0.023). A significantly higher proportion of male patients switched from aspirin + clopidogrel (or any other antiplatelet) and statin to the FDC of clopidogrel and rosuvastatin during treatment, compared to female patients (P=0.041). Patient adherence to all prescribed doses was significantly higher in males compared to females (P=0.031) (Appendix 2).

Physician global evaluations of efficacy and tolerability were rated as 'Excellent' significantly more often in the urban region compared to the rural region (P<0.001 for both) (Appendix 3).

Patients in the uneducated group were significantly more likely to report decreased cost and simplified dosing as key reasons for improved adherence to clopidogrel and rosuvastatin therapy compared to the educated group (P=0.032 and P=0.001, respectively). Physician global evaluation of efficacy was rated as 'Excellent' significantly more often in the educated group compared to the uneducated group (58.71% vs. 48.03%; P=0.028) (Appendix 4).

Better adherence and treatment of dyslipidemia were significantly more common reasons for prescribing treatments among patients with an income of >10 lakh compared to those with an income of <5 lakh and five to 10 lakh (P=0.023 and P=0.047, respectively). Better affordability was a significantly more common reason for prescribing treatments among patients with an income of five to 10 lakh, compared to those with an income of <5 lakh or >10 lakh (P<0.001). A significantly higher proportion of patients with an income of >10 lakh underwent thrombolysis, PCI, or CABG for CAD while on clopidogrel and rosuvastatin treatment, compared to those with incomes of <5 lakh and 5-10 lakh (P=0.018). A significantly higher proportion of patients with an income of five to 10 lakh switched from aspirin + clopidogrel (or any other antiplatelet) and statin to a FDC of clopidogrel and rosuvastatin during treatment, compared to those with incomes of <5 lakh and >10 lakh (P=0.003). Decreased cost and improved symptoms and health outcomes were significantly more common reasons for treatment adherence among patients with an income of >10 lakh, compared to those with lower incomes (P=0.018 and P=0.002, respectively). The physician's global evaluations of both efficacy and tolerability were rated as “Excellent” significantly more often in patients with an income of >10 lakh compared to those with lower incomes (P=0.011 and P<0.001, respectively) (Appendix 5).

Once-daily dosing was significantly more common in patients receiving 10 mg of rosuvastatin compared to those on 20 mg (97.93% vs. 92.68%, P<0.001). The median treatment duration was slightly longer in the 10 mg group than in the 20 mg group (20.00 months vs. 18.00 months; P=0.045). Better affordability was a significantly more common reason for prescribing treatment in patients receiving 20 mg of rosuvastatin compared to those on 10 mg (63.01% vs. 55.07%, P=0.012). A significantly higher proportion of patients receiving 10 mg of rosuvastatin underwent thrombolysis, PCI, or CABG for CAD while on clopidogrel and rosuvastatin treatment, compared to those on 20 mg (P<0.001). A significantly higher proportion of patients receiving 10 mg of rosuvastatin switched from aspirin + clopidogrel (or another antiplatelet) and statin to a FDC of clopidogrel and rosuvastatin during treatment, compared to those receiving 20 mg (P<0.001). Forgetting medication twice a month was reported as a significantly more common reason for non-adherence in patients receiving the 10 mg dose of rosuvastatin compared to those receiving the 20 mg dose (P=0.035). Decreased cost and improved symptoms and health outcomes were significantly more common reasons for treatment adherence in patients receiving the 20 mg dose of rosuvastatin compared to those receiving the 10 mg dose (P<0.001 and P=0.010, respectively). The physician’s global evaluations of both efficacy and tolerability were rated as “Excellent” significantly more common in patients receiving the 20 mg dose of rosuvastatin compared to those receiving the 10 mg dose (P=0.004 and P=0.028, respectively) (Appendix 6).

## Discussion

This study evaluated the adherence to combination therapy with rosuvastatin and clopidogrel in patients with ACS. The results provide important insights into the demographic, clinical characteristics, and treatment adherence in a substantial cohort of 975 patients, shedding light on both patient adherence patterns and the tolerability of the medications.

In the current study, the patient had a median age of 57 years, which corresponds to a common age group for individuals affected by CVD. As people age, the risk and prevalence of CVD increase, including conditions such as hypertension, ischemic heart disease, atrial fibrillation, and heart failure. This trend highlights age-related physiological changes and emphasizes the importance of targeted preventive measures in older populations to address the growing burden of CVD [13]. In the present study, the majority of patients were male, comprising 694 (71.18%) total patients. This finding aligns with the known trend that men experience a steadily increasing risk profile for CVD as they age [14].

The combination therapy of rosuvastatin and clopidogrel is widely accepted for the treatment of CVD [4]. In the present study, adherence to the prescribed doses of these medications was notably high, with 931 (95.49%) patients following their regimen. This is an encouraging result that highlights the potential effectiveness of the intervention. High adherence is particularly important, as non-adherence to cardiovascular medications is a well-documented issue that can lead to increased morbidity, mortality, and healthcare costs [15]. Several factors have contributed to the improved adherence observed in this study. Notably, 672 (68.92%) patients reported reduced treatment costs, and 589 (60.41%) cited simplified dosing schedules as key contributors to their adherence. Additionally, 95.28% of patients were prescribed once-daily dosing regimens, which likely enhanced compliance. Previous research has shown that simpler and less frequent dosing schedules improve adherence across various medications [16], whereas complex dosing regimens often lead to reduced patient compliance [17].

The FDC of rosuvastatin and clopidogrel offers several advantages over the free-equivalent combination of separate tablets. It reduces pill burden, simplifies treatment regimens, and lowers the overall cost of therapy. These factors collectively contribute to improved patient adherence, satisfaction, and clinical outcomes. Studies suggest that FDC therapy can enhance medication compliance by approximately 1.29 times compared to free-equivalent combinations. In India, affordability remains a major barrier to consistent medication use, particularly among individuals with lower income levels. Purchasing two separate drugs, even in generic form, can increase the financial burden. In contrast, FDCs are often more cost-effective than the combined price of individual components, making them a more accessible option [6,18-20]. This improved affordability, along with simplified dosing, may help support better long-term adherence and cardiovascular outcomes. In this study, patients with an annual income exceeding 10 lakhs were significantly more likely to report reduced medication costs and improved symptoms and health outcomes as key factors contributing to better adherence, compared to those with lower income levels.

In the current study, most patients reported having a family history of diabetes. This prevalence is significant, as a family history of diabetes serves as a key risk indicator for developing diabetes itself, which is a well-known risk factor for CVD. Consequently, the presence of a family history of diabetes may also indicate an elevated risk for ASCVD [21]. This correlation highlights the importance of considering familial health history when assessing individual cardiovascular risk and implementing preventive measures. 

In the current study, a high occurrence of prior cardiovascular events, including ACS, was observed in this population. This finding emphasizes the need for secondary prevention strategies to reduce the risk of further cardiovascular incidents [22]. In the present study, the most common risk factors were hypertension observed in 749 (76.82%) patients, and diabetes in 706 (72.41%) patients. This indicates the substantial burden of cardiovascular risk factors within this population. These findings align with prior studies, highlighting the significance of these comorbidities in worsening cardiovascular risk and contributing to the progression of CAD. Specifically, diabetes accelerates the progression of atherosclerosis and is linked to a higher risk of developing ACS, while the presence of hypertension further heightens the risk of CAD [23,24]. The coexistence of hypertension and diabetes presents several challenges that can hinder medication adherence. Managing two chronic conditions simultaneously places substantial cognitive and logistical demands on patients, often resulting in inconsistent use of prescribed therapies. This dual burden may also complicate treatment strategies, as complex therapeutic regimens are frequently required, which may further impact adherence [25]. Given the high prevalence of these comorbidities, there is an urgent need for early, targeted interventions. While lifestyle modifications are a cornerstone of management in patients with both hypertension and diabetes [26], pharmacological therapies are often necessary to achieve optimal blood pressure control and prevent additional complications [27].

Switching treatments is commonly recommended to enhance effectiveness, minimize side effects, or lower costs while maintaining therapeutic efficacy [28]. In the current study, 47.49% of patients switched from a combination of aspirin, clopidogrel, and a statin to a combination of clopidogrel and rosuvastatin during treatment. This switching behavior may reflect clinical considerations aimed at improving tolerability, particularly by reducing aspirin-related gastrointestinal side effects, which are common in elderly patients [29]. Additionally, rosuvastatin offers potent lipid-lowering effects with a favorable safety profile and fewer drug interactions [30], which may further support improved adherence and long-term patient outcomes in real-world practice.

In the present study, only eight (0.82%) patients experienced side effects from clopidogrel and rosuvastatin therapy, with the most commonly reported side effects being nausea and stomach pain. These findings align with a previous study conducted by Gao H et al., which also demonstrated better clinical efficacy with the combination of rosuvastatin and clopidogrel bisulfate. The study reported a lower adverse reaction rate, reinforcing the conclusion that rosuvastatin plus clopidogrel bisulfate is both safe and effective for treating elderly patients with CHD [3]. The favorable safety profile observed may be attributed to the pharmacokinetic properties of rosuvastatin, which, unlike some other statins, is not primarily metabolized by the cytochrome P450 (CYP450) enzyme system. Consequently, this reduces the potential for drug-drug interactions with clopidogrel and helps preserve platelet aggregation function, further supporting the compatibility and clinical safety of this therapeutic regimen [31].

In terms of efficacy, 548 (56.21%) physicians in the present study rated the combination therapy of clopidogrel and rosuvastatin as excellent based on the physicians' global efficacy evaluation for preventing cardiovascular events, reflecting the robust benefits of this treatment in real-world settings. Moreover, based on the physicians' global tolerability evaluation, 549 (56.31%) physicians also rated the tolerability of this combination as excellent. Tolerability is a key factor that significantly influences patients’ perceived quality of life. Good tolerability not only enhances patient comfort and satisfaction but also contributes to better treatment adherence and persistence, ultimately impacting clinical outcomes [32]. In this context, physician-reported excellent tolerability may indirectly reflect a favorable quality of life. Rosuvastatin is a high-potency statin that has demonstrated significant lipid-lowering effects, while clopidogrel is a potent antiplatelet agent, both of which are essential in reducing atherosclerotic risk [3,4].

Limitations

This study has several limitations. Firstly, its retrospective design may introduce bias and limit the ability to draw causal inferences. Secondly, the non-randomized nature of the study could affect the generalizability of the results. Thirdly, it is non-comparative, with no control or comparator group, which restricts the interpretation of outcomes such as adherence and tolerability as being directly attributable to the FDC. These design limitations hinder the ability to establish causality or assess the comparative effectiveness of the FDC. Additionally, there was no specific criterion or standardized risk scoring system used to define 'high risk'; the classification was based on the treating physician’s discretion as recorded in the medical records. Therefore, future prospective, randomized, and comparative studies are needed to confirm these findings and to establish the relative effectiveness and safety of the FDC.

## Conclusions

This study highlights the high adherence and positive outcomes of combination therapy with clopidogrel and rosuvastatin in individuals at high cardiovascular risk. The majority of patients adhered to the prescribed regimen, with improved adherence linked to reduced costs and simplified dosing. The treatment was well-tolerated and received excellent ratings from physicians for both efficacy and tolerability.

## Appendices

Appendix 1 

**Table 4:** Association of age groups with treatment pattern IQR: interquartile range; PCI: percutaneous coronary intervention; CABG: coronary artery bypass graft; CAD: coronary artery disease; FDC: fixed-dose combination.

Parameters	Age groups (years)			P value
	≥25-<50 (N=194)	≥50-<70 (N=680)	≥70 (N=101)	
Rosuvastatin dose, n (%)				0.020
10 mg	106 (54.62)	339 (49.85)	38 (37.62)	
20 mg	88 (45.36)	341 (50.15)	63 (62.38)	
Frequency, n (%)				0.035
Once daily	178 (91.75)	654 (96.18)	97 (96.04)	
Twice daily	16 (8.25)	26 (3.82)	4 (3.96)	
Treatment duration (months), median (IQR)	18.00 (12.00-27.00)	18.00 (13.00-31.00)	20.00 (12.00-62.00)	0.087
Reasons for prescribing treatments, n (%)				
To treat/reduce cardiovascular complications	179 (92.27)	644 (94.71)	91 (90.10)	0.130
Better adherence	110 (56.70)	420 (61.76)	61 (60.40)	0.444
To treat dyslipidemia	112 (19.24)	409 (70.27)	61 (10.48)	0.823
Better affordability	109 (56.19)	413 (60.74)	54 (53.47)	0.252
Patient undergoes thrombolysis/PCI/CABG for CAD while on clopidogrel and rosuvastatin treatment, n (%)	57 (29.38)	286 (42.06)	34 (33.66)	0.003
Patients switch from aspirin + clopidogrel (any other anti-platelet) and statin to FDC clopidogrel and rosuvastatin during treatment, n (%)	85 (43.81)	346 (50.88)	32 (31.68)	0.001
Patient adherence to all prescribed doses, n (%)	184 (94.85)	651 (95.74)	96 (95.05)	0.849
Reason for non-adherence of the doses, n (%)	[n=10]	[n=29]	[n=5]	0.910
Once a week	2 (20.00)	7 (24.14)	1 (20.00)	
Once a month	4 (40.00)	11 (37.93)	1 (20.00)	
Twice a month	3 (30.00)	9 (60.00)	3 (20.00)	
Cost-related nonadherences	1 (10.00)	2 (6.90)	0	
Reasons for treatment adherence with clopidogrel and rosuvastatin, n (%)				
Decreased cost	134 (69.07)	475 (69.85)	63 (62.38)	0.317
Simplified dosing	125 (64.43)	417 (61.32)	47 (46.53)	0.008
Improved symptoms and health outcomes	120 (61.8)	377 (55.44)	52 (51.49)	0.166
Patient experienced any side effects related to clopidogrel and rosuvastatin treatment in last 6 months, n (%)	0	8 (1.18)	0	-
Nausea	0	3 (37.50)	0	-
Stomach pain	0	3 (37.50)	0	-
Extreme fatigue	0	1 (12.50)	0	-
Tiredness	0	1 (12.50)	0	-
Body aches	0	1 (12.50)	0	-
Gastritis	0	1 (12.50)	0	-
Abdomen pain	0	1 (12.50)	0	-
Physician global evaluation of efficacy, n (%)				0.007
Excellent	129 (66.49)	364 (53.53)	55 (54.46)	
Very good	46 (23.71)	260 (38.24)	40 (39.60)	
Good	13 (6.70)	47 (6.91)	5 (4.95)	
Average	6 (3.09)	9 (1.32)	1 (0.99)	
Physician global evaluation of tolerability, n (%)				0.006
Excellent	128 (65.98)	364 (53.97)	54 (53.47)	
Very good	44 (22.68)	257 (37.79)	40 (39.60)	
Good	18 (9.28)	48 (7.06)	7 (6.93)	
Average	4 (2.06)	8 (1.18)	0	

Appendix 2 

**Table 5:** Association of sex with treatment pattern IQR: interquartile range; PCI: percutaneous coronary intervention; CABG: coronary artery bypass graft; CAD: coronary artery disease; FDC: fixed-dose combination.

Parameters	Sex		P value
	Male (N=694)	Female (N=281)	
Rosuvastatin dose, n (%)			0.692
10 mg	341 (49.14)	142 (50.53)	
20 mg	353 (50.86)	139 (49.47)	
Frequency, n (%)			0.675
Once daily	660 (95.10)	269 (95.73)	
Twice daily	34 (4.90)	12 (4.27)	
Treatment duration (months), median (IQR)	18.00 (12.00-30.00)	18.00 (12.00-30.00)	0.765
Reasons for prescribing treatments, n (%)			
To treat/reduce cardiovascular complications	651 (93.80)	263 (93.59)	0.903
Better adherence	417 (60.09)	174 (61.92)	0.595
To treat dyslipidemia	411 (59.22)	171 (60.85)	0.638
Better affordability	406 (58.50)	170 (60.50)	0.566
Patient undergoes thrombolysis/PCI/CABG for CAD while on clopidogrel and rosuvastatin treatment, n (%)	284 (40.92)	93 (33.10)	0.023
Patients switch from aspirin + clopidogrel (any other anti-platelet) and statin to FDC clopidogrel and rosuvastatin during treatment, n (%)	344 (49.57)	119 (42.35)	0.041
Patient adherence to all prescribed doses, n (%)	669 (96.40)	262 (93.24)	0.031
Reason for non-adherence of the doses, n (%)	[n=25]	[n=19]	0.672
Once a week	4 (16.00)	6 (31.58)	
Once a month	10 (40.00)	6 (31.58)	
Twice a month	9 (36.00)	6 (31.58)	
Cost-related nonadherences	2 (8.00)	1 (5.26)	
Reasons for treatment adherence with clopidogrel and rosuvastatin, n (%)			
Decreased cost	475 (68.44)	197 (70.11)	0.611
Simplified dosing	413 (59.51)	176 (62.63)	0.366
Improved symptoms and health outcomes	385 (55.48)	164 (58.36)	0.410
Patient experienced any side effects related to clopidogrel and rosuvastatin treatment in last 6 months, n (%)	4 (0.58)	4 (1.42)	0.184
Nausea	1 (25.00)	2 ((50.00)	-
Stomach pain	2 (50.00)	1 (25.00)	-
Extreme fatigue	1 (25.00)	0	-
Tiredness	1 (25.00)	0	-
Body aches	1 (25.00)	0	-
Gastritis	0	1 (25.00)	-
Abdomen pain	1 (25.00)	0	-
Physician global evaluation of efficacy, n (%)			0.503
Excellent	387 (55.76)	161 (57.30)	
Very good	246 (35.45)	100 (35.59)	
Good	51 (7.35)	14 (4.98)	
Average	10 (1.44)	6 (2.14)	
Physician global evaluation of tolerability, n (%)			0.150
Excellent	376 (54.18)	173 (61.57)	
Very good	256 (75.07)	85 (24.93)	
Good	52 (7.49)	21 (7.47)	
Average	10 (1.44)	2 (0.71)	

Appendix 3 

**Table 6:** Association of place of stay with treatment pattern IQR: interquartile range; PCI: percutaneous coronary intervention; CABG: coronary artery bypass graft; CAD: coronary artery disease; FDC: fixed-dose combination.

Parameters	Place of stay		P value
	Urban (N=727)	Rural (N=248)	
Rosuvastatin dose, n (%)			0.101
10 mg	349 (48.01)	134 (54.03)	
20 mg	378 (51.99)	114 (45.97)	
Frequency, n (%)			0.136
Once daily	697 (95.87)	232 (93.55)	
Twice daily	30 (4.13)	16 (6.45)	
Treatment duration (months), median (IQR)	18.00 (12.00-30.00)	19.00 (12.00-30.00)	0.793
Reasons for prescribing treatments, n (%)			
To treat/reduce cardiovascular complications	677 (93.12)	237 (95.56)	0.170
Better adherence	437 (60.11)	154 (62.10)	0.580
To treat dyslipidemia	436 (59.97)	146 (58.87)	0.760
Better affordability	425 (58.46)	151 (60.89)	0.502
Patient undergoes thrombolysis/PCI/CABG for CAD while on clopidogrel and rosuvastatin treatment, n (%)	274 (37.69)	103 (41.53)	0.283
Patients switch from aspirin + clopidogrel (any other anti-platelet) and statin to FDC clopidogrel and rosuvastatin during treatment, n (%)	342 (47.04)	121 (48.79)	0.634
Patient adherence to all prescribed doses, n (%)	694 (95.46)	237 (95.56)	0.946
Reason for non-adherence of the doses, n (%)	[n=33]	[n=11]	0.378
Once a week	9 (27.27)	1 (9.09)	
Once a month	11 (33.33)	5 (45.45)	
Twice a month	10 (30.30)	5 (45.45)	
Cost-related nonadherences	3 (9.09)	0	
Reasons for treatment adherence with clopidogrel and rosuvastatin, n (%)			
Decreased cost	501 (68.91)	171 (68.95)	0.991
Simplified dosing	430 (59.15)	159 (64.11)	0.167
Improved symptoms and health outcomes	414 (56.95)	135 (54.44)	0.491
Patient experienced any side effects related to clopidogrel and rosuvastatin treatment in last 6 months, n (%)	7 (0.96)	1 (0.40)	0.399
Nausea	3 (42.86)	0	-
Stomach pain	3 (42.86)	0	-
Extreme fatigue	1 (14.29)	0	-
Tiredness	1 (14.29)	0	-
Body aches	1 (14.29)	0	-
Gastritis	0	1 (100.00)	-
Abdomen pain	1 (14.29)	0	-
Physician global evaluation of efficacy, n (%)			<0.001
Excellent	437 (60.11)	111 (44.76)	
Very good	248 (34.11)	98 (39.52)	
Good	35 (4.81)	30 (12.10)	
Average	7 (0.96)	9 (3.63)	
Physician global evaluation of tolerability, n (%)			<0.001
Excellent	427 (58.73)	122 (49.19)	
Very good	256 (35.21)	85 (34.27)	
Good	39 (5.36)	34 (13.71)	
Average	5 (0.69)	7 (2.82)	

Appendix 4 

**Table 7:** Association of education level with treatment pattern IQR: interquartile range; PCI: percutaneous coronary intervention; CABG: coronary artery bypass graft; CAD: coronary artery disease; FDC: fixed-dose combination.

Parameters	Education level		P value
	Educated (N=746)	Uneducated (N=229)	
Rosuvastatin dose, n (%)			0.411
10 mg	375 (50.27)	108 (47.16)	
20 mg	371 (49.73)	121 (52.84)	
Frequency, n (%)			0.027
Once daily	717 (96.11)	212 (92.58)	
Twice daily	29 (3.89)	17 (7.42)	
Treatment duration (months), median (IQR)	18.00 (13.00-32.00)	18.00 (12.00-29.00)	0.104
Reasons for prescribing treatments, n (%)			
To treat/reduce cardiovascular complications	697 (7.42)	217 (94.76)	0.468
Better adherence	444 (59.52)	147 (64.19)	0.205
To treat dyslipidemia	437 (58.58)	145 (63.32)	0.201
Better affordability	444 (59.52)	132 (57.64)	0.614
Patient undergoes thrombolysis/PCI/CABG for CAD while on clopidogrel and rosuvastatin treatment, n (%)	287 (38.47)	90 (39.30)	0.822
Patients switch from aspirin + clopidogrel (any other anti-platelet) and statin to FDC clopidogrel and rosuvastatin during treatment, n (%)	359 (48.12)	104 (45.41)	0.473
Patient adherence to all prescribed doses, n (%)	713 (95.58)	218 (95.20)	0.809
Reason for non-adherence of the doses, n (%)	[n=33]	[n=11]	0.494
Once a week,	6 (18.18)	4 (36.36)	
Once a month	12 (36.36)	4 (36.36)	
Twice a month	12 (36.36)	3 (27.27)	
Cost-related nonadherences	3 (9.09)	0	
Reasons for treatment adherence with clopidogrel and rosuvastatin, n (%)			
Decreased cost	501 (67.16)	171 (74.67)	0.032
Simplified dosing	429 (57.51)	160 (69.87)	0.001
Improved symptoms and health outcomes	412 (55.23)	137 (59.83)	0.220
Patient experienced any side effects related to clopidogrel and rosuvastatin treatment in last 6 months, n (%)	6 (0.80)	2 (0.87)	0.919
Nausea	3 (50.00)	0	-
Stomach pain	2 (33.33)	1 (50.00)	-
Extreme fatigue	1 (16.67)	0	-
Tiredness	1 (16.67)	0	-
Body aches	1 (16.67)	0	-
Gastritis	0	1 (50.00)	-
Abdomen pain	1 (16.67)	0	-
Physician global evaluation of efficacy, n (%)			0.028
Excellent	438 (58.71)	110 (48.03)	
Very good	253 (33.91)	93 (40.61)	
Good	44 (5.90)	21 (9.17)	
Average	11 (1.47)	5 (2.18)	
Physician global evaluation of tolerability, n (%)			0.437
Excellent	430 (57.64)	119 (51.97)	
Very good	255 (34.18)	86 (37.55)	
Good	52 (6.97)	21 (9.17)	
Average	9 (1.21)	3 (1.31)	

Appendix 5 

**Table 8:** Association of income with treatment pattern IQR: interquartile range; PCI: percutaneous coronary intervention; CABG: coronary artery bypass graft; CAD: coronary artery disease; FDC: fixed-dose combination.

Parameters	Income (Lakh)			P value
	<5 (N=385)	5-10 (N=313)	>10 (N=76)	
Rosuvastatin dose, n (%)				0.062
10 mg	193 (50.13)	162 (51.76)	28 (36.84)	
20 mg	192 (49.87)	151 (48.24)	48 (63.16)	
Frequency, n (%)				0.289
Once daily	365 (94.81)	304 (97.12)	72 (94.74)	
Twice daily	20 (5.19)	9 (2.88)	4 (5.26)	
Treatment duration (months), median (IQR)	18.00 (12.00-30.00)	20.00 (13.00-29.00)	21.00 (13.00-35.75)	0.401
Reasons for prescribing treatments, n (%)				
To treat/reduce cardiovascular complications	361 (49.72)	290 (39.94)	75 (10.33)	0.148
Better adherence	214 (55.58)	198 (63.26)	53 (69.74)	0.023
To treat dyslipidemia	215 (55.84)	185 (59.11)	54 (71.05)	0.047
Better affordability	191 (49.61)	215 (68.69)	45 (59.21)	<0.001
Patient undergoes thrombolysis/PCI/CABG for CAD while on clopidogrel and rosuvastatin treatment, n (%)	133 (34.55)	134 (42.81)	37 (48.68)	0.018
Patients switch from aspirin + clopidogrel (any other anti-platelet) and statin to FDC clopidogrel and rosuvastatin during treatment, n (%)	167 (43.38)	176 (56.23)	37 (48.68)	0.003
Patient adherence to all prescribed doses, n (%)	364 (94.55)	301 (96.17)	74 (97.37)	0.417
Reason for non-adherence of the doses, n (%)	[n=21]	[n=12]	[n=2]	
Once a week	6 (28.57)	1 (8.33)	1 (50.00)	0.696
Once a month	7 (33.33)	4 (33.33)	1 (50.00)	
Twice a month	7 (33.33)	6 (50.00)	0	
Cost-related nonadherences	1 (4.76)	1 (8.33)	0	
Reasons for treatment adherence with clopidogrel and rosuvastatin, n (%)				
Decreased cost	250 (64.94)	232 (74.12)	57 (75.00)	0.018
Simplified dosing	215 (55.84)	202 (64.54)	49 (64.47)	0.048
Improved symptoms and health outcomes	195 (50.65)	177 (56.55)	55 (72.37)	0.002
Patient experienced any side effects related to clopidogrel and rosuvastatin treatment in last 6 months, n (%)	3 (0.78)	3 (0.96)	1 (1.32)	0.895
Nausea	1 (33.33)	2 (66.67)	0	-
Stomach pain	2 (66.67)	1 (33.33)	0	-
Extreme fatigue	0	0	1 (100.00)	-
Tiredness	0	0	1 (100.00)	-
Body aches	0	0	1 (100.00)	-
Gastritis	0	0	0	-
Abdomen pain	0	1 (33.33)	0	-
Physician global evaluation of efficacy, n (%)				0.011
Excellent	215 (55.84)	169 (53.99)	52 (68.42)	
Very good	126 (32.73)	127 (40.58)	21 (27.63)	
Good	35 (9.09)	14 (4.47)	3 (3.95)	
Average	9 (2.34)	3 (0.96)	0	
Physician global evaluation of tolerability, n (%)				<0.001
Excellent	218 (56.62)	164 (52.40)	48 (63.16)	
Very good	117 (30.39)	136 (43.45)	25 (32.89)	
Good	41 (10.65)	12 (3.83)	3 (3.95)	
Average	9 (2.34)	1 (0.32)	0	

Appendix 6 

**Table 9:** Association of rosuvastatin dose with treatment pattern IQR: interquartile range; PCI: percutaneous coronary intervention; CABG: coronary artery bypass graft; CAD: coronary artery disease; FDC: fixed-dose combination.

Parameters	Rosuvastatin dose		P value
	10 mg (N=483)	20 mg (N=492)	
Frequency, n (%)			<0.001
Once daily	473 (97.93)	456 (92.68)	
Twice daily	10 (2.07)	36 (7.32)	
Treatment duration (months), median (IQR)	20.00 (13.00-30.00)	18.00 (12.00-30.00)	0.045
Reasons for prescribing treatments, n (%)			
To treat/reduce cardiovascular complications	446 (92.34)	468 (95.12)	0.073
Better adherence	285 (59.01)	306 (62.20)	0.308
To treat dyslipidemia	274 (56.73)	308 (62.60)	0.062
Better affordability	266 (55.07)	310 (63.01)	0.012
Patient undergoes thrombolysis/PCI/CABG for CAD while on clopidogrel and rosuvastatin treatment, n (%)	218 (45.13)	159 (32.32)	<0.001
Patients switch from aspirin + clopidogrel (any other anti-platelet) and statin to FDC clopidogrel and rosuvastatin during treatment, n (%)	258 (53.42)	205 (41.67)	<0.001
Patient adherence to all prescribed doses, n (%)	465 (96.27)	466 (94.72)	0.241
Reason for non-adherence of the doses, n (%)	[n=18]	[n=26]	0.035
Once a week	6 (33.33)	4 (15.38)	
Once a month	6 (33.33)	10 (38.46)	
Twice a month	3 (16.67)	12 (46.15)	
Cost-related nonadherences	3 (16.67)	0	
Reasons for treatment adherence with clopidogrel and rosuvastatin, n (%)			
Decreased cost	305 (63.15)	367 (74.59)	<0.001
Simplified dosing	293 (60.66)	296 (60.163)	0.873
Improved symptoms and health outcomes	252 (52.17)	297 (60.37)	0.010
Patient experienced any side effects related to clopidogrel and rosuvastatin treatment in last 6 months, n (%)	4 (0.83)	4 (0.81)	0.979
Nausea	2 (50.00)	1 (25.00)	-
Stomach pain	1 (25.00)	2 (50.00)	-
Extreme fatigue	1 (25.00)	0	-
Tiredness	1 (25.00)	0	-
Body aches	1 (25.00)	0	-
Gastritis	0	1 (25.00)	-
Abdomen pain	0	1 (25.00)	-
Physician global evaluation of efficacy, n (%)			0.004
Excellent	250 (51.76)	298 (60.57)	
Very good	180 (37.27)	166 (33.74)	
Good	41 (8.49)	24 (4.88)	
Average	12 (2.48)	4 (0.81)	
Physician global evaluation of tolerability, n (%)			0.028
Excellent	259 (53.62)	290 (58.94)	
Very good	169 (34.99)	172 (34.96)	
Good	47 (9.73)	26 (5.28)	
Average	8 (1.66)	4 (0.81)	
